# Mathematical model predicts tumor control patterns induced by fast and slow cytotoxic T lymphocyte killing mechanisms

**DOI:** 10.1038/s41598-023-49467-6

**Published:** 2023-12-18

**Authors:** Yixuan Wang, Daniel R Bergman, Erica Trujillo, Alexander T. Pearson, Randy F. Sweis, Trachette L. Jackson

**Affiliations:** 1 Department of Mathematics, University of Michigan, Ann Arbor, MI 48109 USA; 2 Department of Medicine, Section of Hematology/Oncology, The University of Chicago, Chicago, IL 60637 USA

**Keywords:** Tumour heterogeneity, Tumour immunology, Cancer models, Computer modelling, Differential equations

## Abstract

Immunotherapy has dramatically transformed the cancer treatment landscape largely due to the efficacy of immune checkpoint inhibitors (ICIs). Although ICIs have shown promising results for many patients, the low response rates in many cancers highlight the ongoing challenges in cancer treatment. Cytotoxic T lymphocytes (CTLs) execute their cell-killing function via two distinct mechanisms: a fast-acting, perforin-mediated process and a slower, Fas ligand (FasL)-driven pathway. Evidence also suggests that the preferred killing mechanism of CTLs depends on the antigenicity of tumor cells. To determine the critical factors affecting responses to ICIs, we construct an ordinary differential equation model describing in vivo tumor-immune dynamics in the presence of active or blocked PD-1/PD-L1 immune checkpoint. Specifically, we identify important aspects of the tumor-immune landscape that affect tumor size and composition in the short and long term. We also generate a virtual cohort of mice with diverse tumor and immune attributes to simulate the outcomes of immune checkpoint blockade in a heterogeneous population. By identifying key tumor and immune characteristics associated with tumor elimination, dormancy, and escape, we predict which fraction of a population potentially responds well to ICIs and ways to enhance therapeutic outcomes with combination therapy.

## Introduction

Immunotherapy has remarkably improved outcomes for many cancer patients. To have efficacy, immunotherapies must overcome immunosuppression induced by tumors and their microenvironment to allow the cytotoxic immune cells to target and kill cancer cells^1^. Immune checkpoint inhibitors (ICIs) are a well-studied class of immunotherapeutics that revitalize the killing capacity of immune cells by blocking the activation of inhibitory immunoreceptors^2^. Immune checkpoint blockade therapy often results in a more durable response than chemotherapy or targeted therapies^2^ and has shown remarkable results for many patients. However, the low overall response rates in many cancers present an ongoing challenge to clinicians. For example, objective response to checkpoint blockade monotherapy remains near 20% in patients with bladder cancer^3^. Over the past decade, there has been keen interest in research to improve the efficacy of blocking the programmed death-1/programmed death-ligand 1 (PD-1/PD-L1) immune checkpoint^4^. The FDA has approved seven monoclonal antibodies that target the PD-1/PD-L1 checkpoint with supplemental indications in over fifteen cancer types and two tissue-agnostic conditions^5^.

Adding further complexity to the antitumor immune responses is the fact that Cytotoxic T lymphocytes (CTLs) execute their cell-killing function via at least two distinct mechanisms. The first process is fast-acting and perforin/granzyme mediated^6^. CTL-derived granzymes enter the tumor cell through perforin pores to induce structural damage and thus apoptosis^7^. The second process is a slower, FasL-driven killing mechanism^6^. FasL, a type II transmembrane protein upregulated on CTLs, can engage Fas on the target cell to trigger signaling that causes the activation of the apoptotic cascade^8^. In one study, perforin-based killing was detected within thirty minutes, whereas FasL-based killing was detected no sooner than two hours after tumor cell conjugated with CTL^6^ . More recently, Cassioli and Baldari corroborated that granzyme/perforin mediated killing happens faster than Fas-dependent killing^8^. There is incomplete data regarding the manifestations of contact vs. granule-based T cell killing in solid tumors; however, some evidence suggests that CTLs switch from fast to slow killing with decreased antigen load^6^, demonstrating different behaviors of the immune system towards tumor cells with varying antigenicity. The antigenicity of a tumor can be defined as the extent to which tumor cells display HLA-restricted antigens that can be selectively or specifically recognized by immune cells^9^. One of the immunoevasive strategies cancer cells employ is the downregulation or loss of antigens, which can occur due to the immune selection of cancer cells that lack immunogenic tumor antigens and through the acquisition of defects in antigen presentation^10^. Loss of antigenicity impairs the ability of natural immune responses to control cancers, impedes immunotherapies that work by re-invigorating CTLs, and potentially alters future responsiveness to additional treatments^11^.

By constructing and analyzing data-driven mathematical and computational models, we aim to investigate the roles of antigenicity, differential immune cell-kill mechanisms and other aspects of the tumor-immune landscape in immune checkpoint blockade therapy, and to formulate therapeutic strategies to enhance outcomes. The complex interactions between tumor and the immune system result in vastly different outcomes such as tumor elimination, tumor dormancy and uncontrolled tumor growth. One way to predict the specific circumstances leading to these fates is through non-linear ordinary differential equations (ODEs), which model cellular interactions and reveal the temporal dynamics of the components in tumor-immune interactions. In what has become a classic model of tumor immune dynamics, Kuzentzov proposed a system of ODEs that model the CTL response to the growth of an immunogenic tumor^12^. Key features of this model include the characterization of antigen-independent and antigen-stimulated recruitment of cytotoxic effector cells to the tumor site, and an immune-mediated death rate of tumor cells that increases proportionally as the number of T cells increases. Alternative mathematical descriptions of tumor cell kill, such as the “Beddington” functional response^13^, have now been proposed to reflect the more realistic assumption that immune cell killing saturates as the number of immune cells increases.

In this study, we test the hypothesis that immune checkpoint therapy impacts both the total tumor volume and the proportion of the two tumor cell phenotypes due to differential killing mechanisms associated with each tumor phenotype. Our analysis highlights important parameters that affect the outcomes of immune checkpoint blockade. Among these, some parameters characterize the tumor-immune landscape, and others can be modulated by cancer treatments such as chemotherapy and additional types of immunotherapy. Understanding the impact of different parameters on tumor volume and phenotypic composition enables us to gain insights into which patients are most likely to respond to ICIs and what combination therapy can result in better therapeutic outcomes.

## Methods

**Figure 1:**
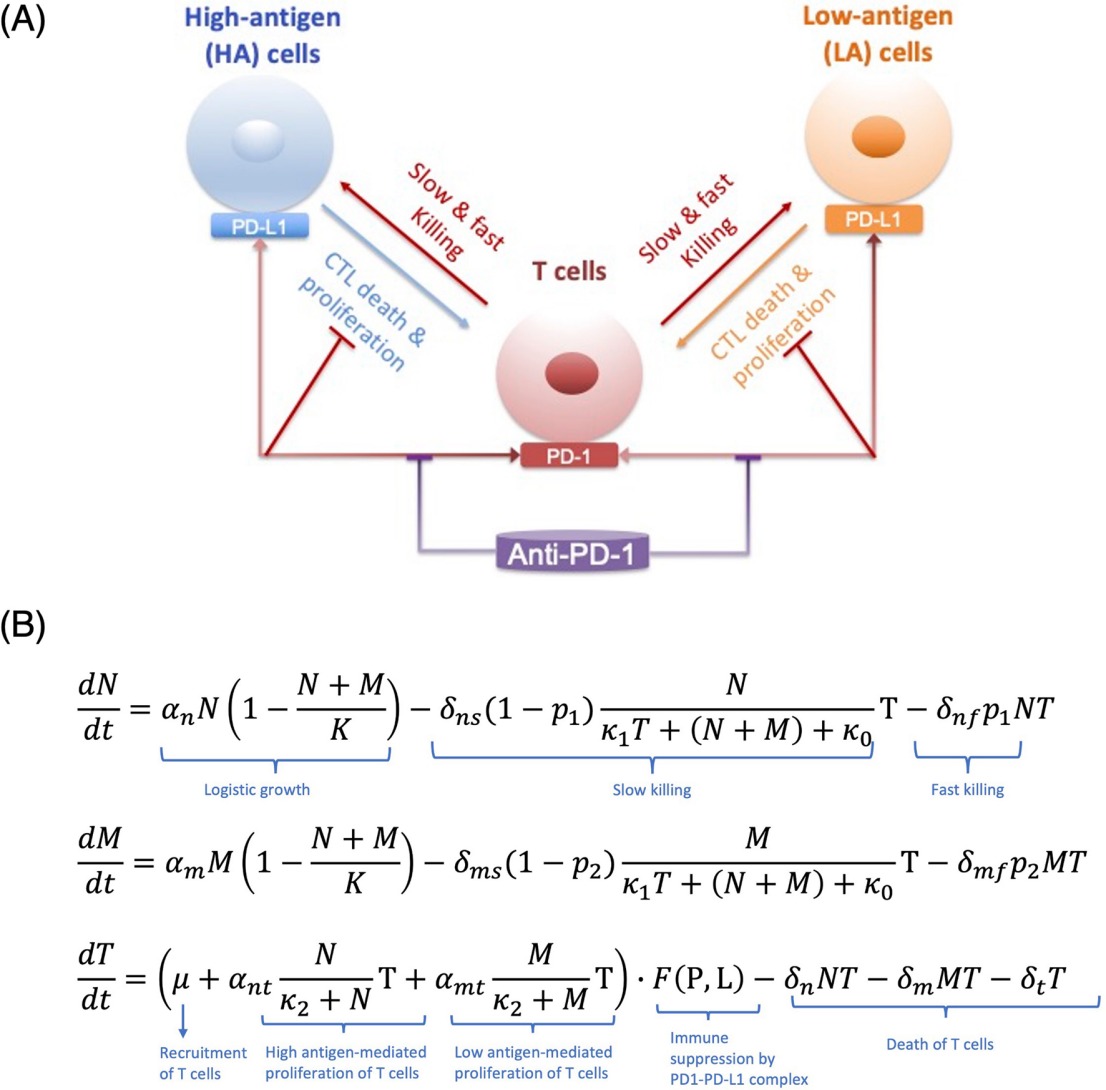
(**A**) Schematic diagram and equations of the ODE model describing tumor-immune dynamics. The two tumor cell phenotypes are high antigen (N) cells and low antigen (M) cells. T cells kill tumor cells via two mechanisms: a fast-acting and perforin/granzyme-mediated process, and a slower, Fas ligand (FasL)-driven process. PD-1 expressed on T cells and PD-L1 expressed on tumor cells interact to inhibit T cell activity in tumor killing. Anti-PD-1 prevents the engagement of PD-1 and PD-L1. (**B**) Model equations.

Our mathematical model reflects the current biological understanding of tumor-immune interactions including the role of the PD-1/PD-L1 immune checkpoint. We develop a “checkpoint active” model that describes the fully functional PD-1/PD-L1 signaling pathway leading to immune suppression. We then relax this assumption by removing the immunosuppressive effects by the PD-1/PD-L1 complex to formulate a model with the PD-1/PD-L1 signal inhibited to study the implications of a perfect checkpoint blockade therapy. The model captures the temporal evolution of the number of high antigen tumor cells (*N*), low antigen tumor cells (*M*) and cytotoxic T cells (*T*). Model variables and their units are described in Table 1, S1. Figure 1A is a schematic diagram of the components of the ODE model, with equations given in Fig. 1B. Parameters that have significant impact on post treatment tumor size and composition include: the probability of high ($p_1$) and low ($p_2$) antigen tumor cell death via fast killing; the rates of high ($\delta _{nf}$) and low ($\delta _{mf}$) tumor cell death via fast killing; the rate of T cell recruitment to the tumor site, $\mu$; and the maximum rate of additional antigen-stimulated T cell proliferation by high ($\alpha _{nt}$) and low($\alpha _{mt}$) antigen tumor cells. Descriptions of all parameters, their units and baseline values parametrized for mice are included in Table 1. A detailed description of the mathematical model is in S1.

**Table 1:** Baseline parameters.

Parameter	Description	Value (baseline)	Units	Source
$\alpha _n$	Proliferation rate of high antigen tumor cells	0.05–0.6 (0.337)	per day	Estimated^14^
$\alpha _m$	Proliferation rate of low antigen tumor cells	0.05–0.6 (0.337)	per day	Estimated^14^
K	Carrying capacity for tumor cells	$3{-}6 \cdot 10^9 (5 \cdot 10^9$)	# of cells	Estimated
$\delta _{ns}$	Maximum CTL-induced death rate of high antigen tumor cells via the slow killing mechanism	1–12 (4)	per day	Estimated^15^
$\delta _{ms}$	Maximum CTL-induced death rate of low antigen tumor cells via the slow killing mechanism	1–12 (4)	per day	Estimated^15^
$\delta _{nf}$	CTL-induced death rate of high antigen tumor cells via the fast-killing mechanism	$10^{-8}{-}10^{-6} (2.5 \cdot 10^{-7})$	per cell per day	Estimated^14^
$\delta _{mf}$	CTL-induced death rate of low antigen tumor cells via the fast-killing mechanism	$10^{-8}{-}10^{-6} (2.5 \cdot 10^{-7})$	per cell per day	Estimated^14^
$\kappa _0$	Half-saturation constant in maximum CTL-induced death rate of via the slow killing mechanism	$10^6{-}10^8(2 \cdot 10^7)$	# of cells	Estimated
$\kappa _1$	Saturation effect by immune cells on slow killing	0.01–1 (0.5)	Dimensionless	Estimated^12^
$\kappa _2$	Half-saturation constant in the antigen-mediated T cell proliferation rate	$10^6{-}10^8 (2.019 \cdot 10^7)$	# of cells	Estimated^14^
$\delta _t$	Death rate of T cells	0–0.5 (0.0412)	per day	^12,16^
$\mu$	Activation and recruitment rate of T cells	$5 \cdot 10^3{-}1.5 \cdot 10^5 (2 \cdot 10^4)$	# per day	Estimated
$\delta _n$	CTL death rate due to interactions with high antigen tumor cells	$10^{-11}{-}10^{-9} (3.422 \cdot 10^{-10})$	per cell per day	Estimated^12^
$\delta _m$	CTL death rate due to interactions with low antigen tumor cells	$10^{-11}{-}10^{-9} (3.422 \cdot 10^{-10})$	per cell per day	Estimated^12^
$p_1$	Probability of high antigen tumor cells death via the fast-killing mechanism	0–1 (0.92)	Dimensionless	Estimated
$p_2$	Probability of low antigen tumor cells death via the fast-killing mechanism	0–1 (0.33)	Dimensionless	Estimated
$\alpha _{nt}$	Maximum rate of CTL proliferation activated by high antigen tumor cells	0–0.5 (0.15)	per day	Estimated^12,14,16^
$\alpha _{mt}$	Maximum rate of CTL proliferation activated by low antigen tumor cells	0–0.5 (0.15)	per day	Estimated^12,14,16^
$\rho _p$	Molar concentration of PD-1 per T cell	$10^{-12}{-}10^{-10} (1.259 \cdot 10^{-11})$	μM	^16,17^
$\rho _l$	Molar concentration of PD-L1 per T cell	$10^{-12}{-}2 \cdot 10^{-10} (2.510 \cdot 10^{-11})$	μM	^16,17^
$\epsilon _c$	Expression of PD-L1 on tumor cells vs. T cells	1–50 (10)	Dimensionless	^17^
$\mu _{PA}$	Blocking rate of PD-1 by anti-PD-1	6.45–273 (8.945)	L/μ mol/h	^17,18^
$k_{TQ}$	Inhibition of T cells by PD-1/PD-L1	$10^{-10}{-}10^{-8} (1.296 \cdot 10^{-9})$	(μM)^2^	^17,18^

## Results

### Impact of immune responses parameters on long-term tumor volume and composition after complete checkpoint blockade

Many parameters of our model are crucial because they either characterize the distinct immune cell-kill mechanisms or can be modulated with therapy. By changing some of these parameters, we now study the impact of the different cell-kill mechanisms ($p_1,p_2$) and individual or combined therapies. Examples of treatment strategies include immune checkpoint inhibition ($F(P,L) = 1$), stimulated immune cell expansion due to the administration of therapeutic cytokines like IL-2 (increasing in $\alpha _{nt},\alpha _{mt}$)^19^, and adoptive T cell transfer (increasing $\mu$). We conducted global sensitivity analysis (S2) to determine which model parameters have the greatest impact on tumor growth. With checkpoint active, the tumor growth is only sensitive to intrinsic properties of the tumor such as proliferation rates. With checkpoint completely blocked, tumor volume and composition are largely influenced by immune parameters, including the probability of fast killing ($p_1, p_2$), differential immune stimulation by high or low antigen tumor cells ($\alpha _{nt}, \alpha _{mt}$) and the immune effector cell localization rate ($\mu$) as shown in (Fig. S1). Therefore, we focus on model predictions when these parameters vary.

We use bifurcation analysis to investigate how stable steady state tumor volume and composition change in response to variations in sensitive parameters when the PD-1/PD-L1 checkpoint is completely blocked. We plot two and three-parameter bifurcation diagrams to reveal important relationships between parameters associated with T cell killing mechanism ($p_1, p_2$) and those that can be modulated by treatment ($\mu , \alpha _{nt}, \alpha _{mt}$).

**Figure 2:**
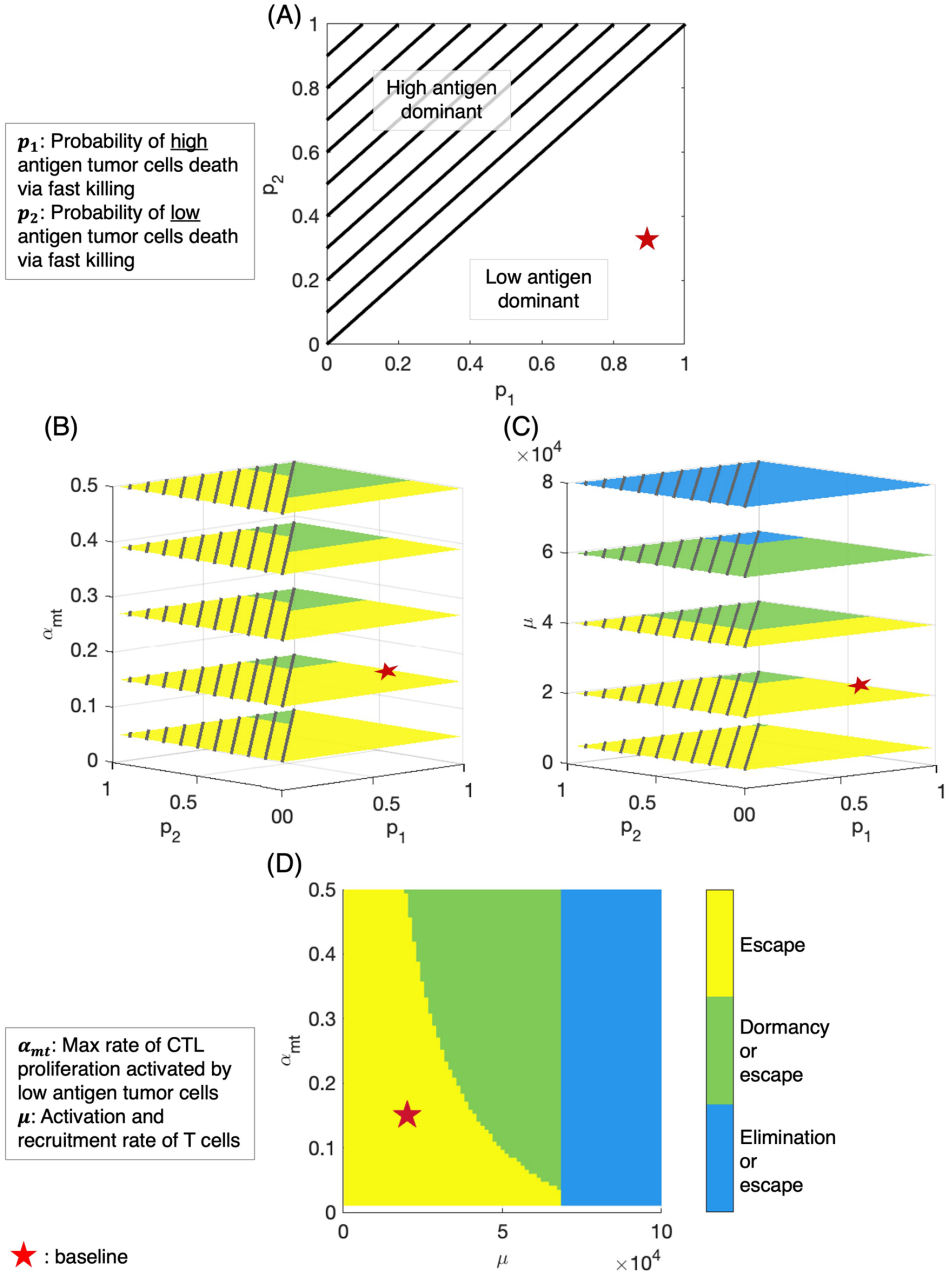
Bifurcation analysis illustrates important variations in steady state tumor size, composition, and response to ICI monotherapy or combination therapy. (**A**) Steady state tumor composition in relation to $p_1, p_2$ values. High (low) antigen dominant: consisting of only high (low) antigen tumor cells. (**B**) Response to changing $\alpha _{mt}$ (e.g. cytokine therapy) at various $p_1, p_2$ values. Two outcomes in terms of tumor size: escape (yellow) and bistability with dormancy or escape (green). (**C**) Response to changing $\mu$ (e.g. adoptive T cell transfer) at various $p_1, p_2$ values. One more outcome in addition to (**B**): bistability with elimination or escape (blue). (**D**) Comparison of the impact of $\mu$ and $\alpha _{mt}$ on steady state tumor size. Escape: tumor size $\ge 500\,\text {mm}^{3}$; dormancy: $0<$ tumor size $< 500\,\text {mm}^{3}$; elimination: tumor size $=0 \,\text {mm}^{3}$. Red star: baseline parameters. Parameters not shown are fixed at baseline values.

#### Differential immune cell-kill mechanisms determine long-term tumor composition

Figure 2A shows how tumor composition in a tumor-persistent steady state varies depending on the values of $p_1$ and $p_2$. When a tumor of any size persists at steady state, the $p_1 - p_2$ parameter space is dissected into regions where the tumor consists of either only high or only low antigen tumor cells, represented by striped and solid regions, respectively. These high antigen-dominant and low antigen-dominant scenarios are the only two types of nonzero steady state outcomes for tumor composition predicted by our model for the parameter ranges in Table 1. Figure 2A shows that when the probability of high antigen tumor cell death via the fast-killing mechanism is greater than that for low antigen tumor cells ($p_1 > p_2$), the final tumor composition at steady steady will be homogeneous and only consists of low antigen tumor cells, and vice versa.

#### Combination checkpoint blockade and cytokine therapy enhances opportunities for tumor dormancy

Our model predicts three types of outcomes in terms of steady state tumor size: elimination, dormancy, and escape. Elimination is defined as no tumor cells present. Dormancy is characterized by the tumor approaching a small, nonzero steady state, and escape is characterized by the tumor approaching carrying capacity. In addition, our model also predicts bistability, which occurs when the steady state tumor can be any two among the three outcomes (e.g. elimination or dormancy) depending on the initial conditions.

Fixing other parameters at baseline values, we explored the impact of $p_1, p_2$, and $\alpha _{mt}$ on the realization of these outcomes. Figure 2B considers the $p_1 - p_2$ bifurcation plane for five values of $\alpha _{mt}$, the maximum rate of CTL proliferation activated by low antigen tumor cells. The slices are taken at $\alpha _{mt} =$ 0.05, 0.15, 0.27, 0.39, 0.5, with the second from the bottom slice representing our baseline $\alpha _{mt}$. This figure shows that there are two types of therapeutic outcomes in terms of steady state tumor size: escape and bistability, represented by yellow and green, regions, respectively. The bistability region in this case includes both dormancy and escape as possible outcomes. With baseline parameters (red star in Fig. 2B) and the PD-1/PD-L1 checkpoint blocked, the tumor will escape and grow to carrying capacity. However, an additional therapy (e.g. cytokine therapy) that increases $\alpha _{mt}$ makes tumor dormancy possible. More generally, our model predicts that a therapy that modulates $\alpha _{mt}$ shrinks the region of $p_1 - p_2$ values where the tumor escapes with certainty and expands the region where the tumor can potentially stabilize in a small, dormant state. It is noted that steady state tumors in Fig. 2B will be either very large (escape) or small (dormant) and either completely high ($p_1 < p_2$) or low ($p_1 > p_2$) antigen dominant. One would observe the same trend if $\alpha _{nt}$ is used instead of $\alpha _{mt}$ because high and low antigen tumor cells are modelled in the same way mathematically.

#### Combination checkpoint blockade and adoptive T cell therapy enhances opportunities for tumor elimination

Similarly, Fig. 2C considers the $p_1 - p_2$ bifurcation plane for five values of $\mu$, the T cell recruitment and activation rate. The slices are taken at $\mu = (0.5, 2, 4, 6, 8) \times 10^4$, with the second from the bottom slice representing our baseline $\mu$. This figure again shows that the yellow region, representing escape, shrinks as $\mu$ increases. However, as $\mu$ increases further, a third outcome emerges, bistability resulting in elimination or escape represented by the blue regions in Fig. 2C. These results imply that an additional therapy that increases $\mu$ (e.g. adoptive T cell therapy) can potentially, result in tumor clearance. With baseline parameter values (red star in Fig. 2C) and the PD-1/PD-L1 checkpoint completely blocked, the tumor will grow to carrying capacity. In this case, increasing $\mu$ can result in tumor dormancy or even elimination. Overall, increasing $\mu$ shrinks the region of $p_1 - p_2$ values where the tumor escapes with certainty and expands the region where the tumor has a chance of being eliminated or stabilizing in a small, dormant state.

#### Pre-treatment immune landscape determines the feasibility of combination therapeutic strategies

Taken together, the results lead us to investigate the potential of combining cytokine and adoptive T cell therapies by exploring the $\mu - \alpha _{mt}$ plane. Figure 2D shows how this type of combination therapy impacts therapeutic outcomes when $p_1,p_2$ are fixed at their baseline values. Here we see that if a tumor is characterized by small intrinsic values of $\mu$ and $\alpha _{mt}$ that would normally lead to escape (the lower left corner of the graph), the fastest way to move to a better outcome with the least amount of additional treatments is combination therapy (checkpoint blockade, adoptive T cell transfer and cytokine therapy) that simultaneously increases both $\mu$ and $\alpha _{mt}$. Figure 2D also implies that it is now possible with combination therapy (checkpoint blockade and adoptive T cell therapy) to only increase $\mu$ and move from any location in the yellow escape region to the blue region where elimination is possible. If a tumor is characterized by our baseline parameter values represented by the red star in Fig. 2D, then additional cytokine therapy within the range for $\alpha _{mt}$ that we consider will be ineffective for changing the therapeutic outcome of escape, but if $\alpha _{mt}$ can be increased to approximately four times our baseline value, dormancy is possible.

### Clinical implications of variable probabilities of fast killing

**Figure 3:**
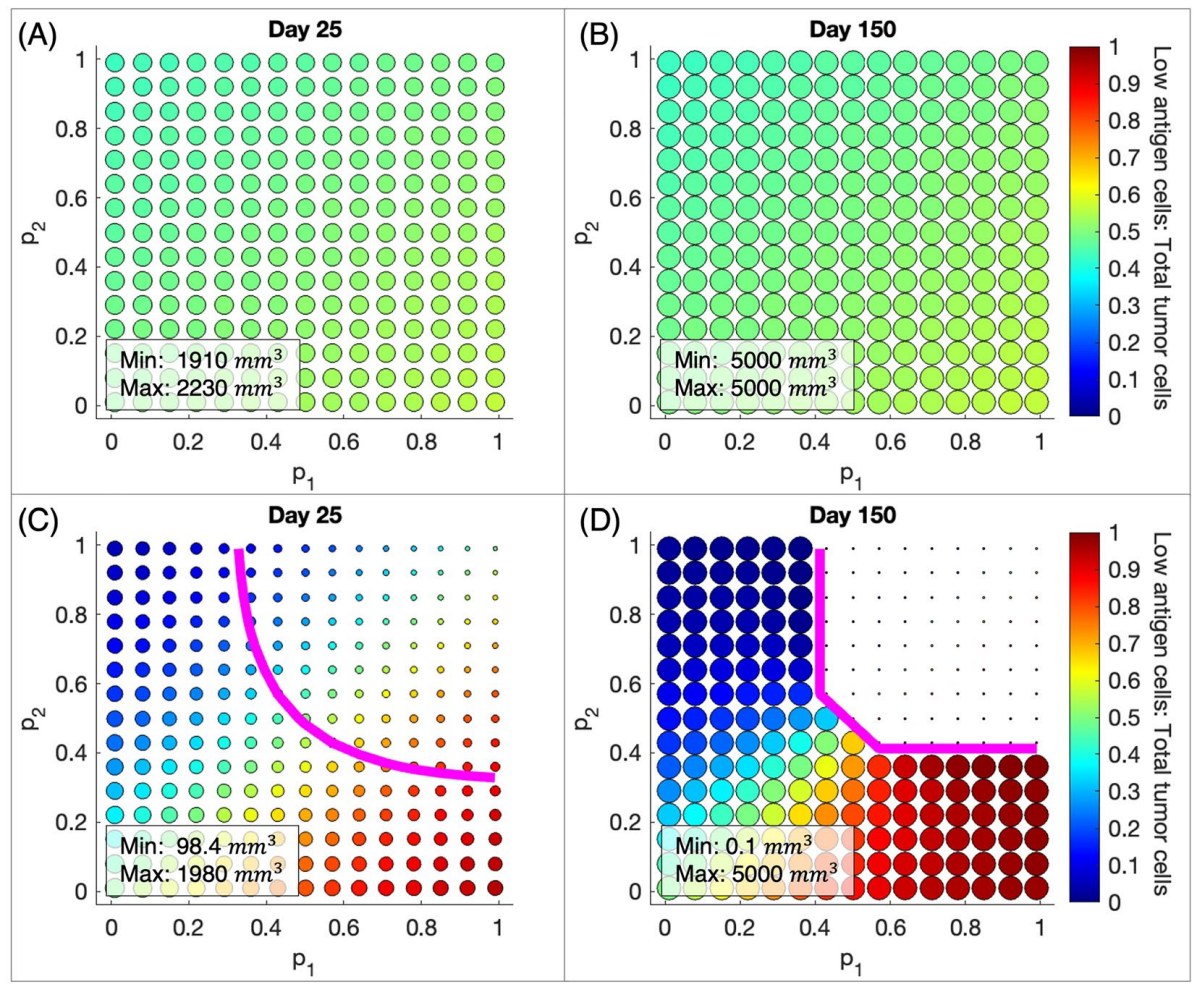
Probability of “fast” T cell killing ($p_1, p_2$) determines the dominant cell type and tumor size in response to checkpoint blockade therapy. (**A**,**B**) Tumor size and composition with checkpoint active on Day 25 and Day 150. (**C**,**D**) Tumor size and composition with checkpoint blocked on Day 25 and Day 150. Magenta line: combinations of $p_1, p_2$ that result in $75\%$ reduction in total tumor volume after checkpoint blockade, relative to when the checkpoint is active. Size of the circles: tumor size; color of circles: the ratio of low antigen tumor cells to total tumor cells, a measure of tumor composition. Initial tumor size in all simulations is $1\,\text {mm}^{3}$ and consists of 50% high antigen tumor cells and 50% low antigen tumor cells.

The previous subsection examined how steady state tumor sizes vary with parameters that can be modulated by therapy as the probability of tumor cell death via fast killing changes. These steady states are rarely seen in practice. To consider these effects on a clinically relevant timeline, we measure the total tumor volume on Day 25 and Day 150. The first end point of 25 days was chosen because model tumor xenograft experiments in mice are often conducted for 3–4 weeks. The second endpoint of 150 days was chosen to represent the timescale of clinical elimination. Figure 3 shows the changes in total tumor volume and composition on Day 25 and Day 150 for varying $p_1, p_2$ combinations when the PD-1/PD-L1 checkpoint is active or completely blocked. Again, $p_1 (p_2)$ is the probability of high (low) antigen tumor cell death via the fast-killing mechanism. Due to limitations of current imaging technology, tumors smaller than $0.1\,\text {mm}^{3}$ cannot be detected^20^. Therefore, we define clinical elimination as a tumor smaller than $0.1\,\text {mm}^{3}$. For the same reason, we stop our simulations when the total tumor size reaches $0.1\,\text {mm}^{3}$ or below. When this happens, the tumor size remains at $0.1\,\text {mm}^{3}$. The size of a circle in Fig. 3 represents the size of the tumor and the color of a circle is a measure of tumor composition as it represents the ratio of low antigen tumor cells to total tumor cells. As the tumors move up the color bar from blue to red, they transition from high antigen-dominant to low antigen-dominant.

Figure 3A shows that as $p_1, p_2$ vary when the checkpoint is active, the total tumor volume on Day 25 remains largely unaffected; all tumors are within about $300\,\text {mm}^{3}$ of each other. By Day 150, these tumors have all reached the maximum carrying capacity volume (Fig. 3B). Tumor composition varies little when the checkpoint is active despite large variations in $p_1, p_2$. The initial tumor size is $1 \,\text {mm}^{3}$ and consists of 50% high antigen tumor cells and 50% low antigen tumor cells. As the difference between $p_1$ and $p_2$ increases, moving towards the lower right corner, there is a modest shift toward low antigen dominance with low antigen tumor cells making up at most 56% of the tumor cells. Similarly, moving towards the upper left corner, there is a modest shift toward high antigen dominance.

Outcomes for complete checkpoint blockade are shown in Fig. 3C and D. When $p_1 > p_2$, the proportion of low antigen tumor cells in the tumor increases significantly after checkpoint blockade, as illustrated by the shift to more red colors in the lower right portion of the graphs. The magenta curve in Fig. 3C and D represents the combinations of $p_1, p_2$ that result in $75\%$ reduction in total tumor volume and our baseline values fall into this region. These figures demonstrate the importance of the probability of fast killing on the therapeutic outcomes of PD-1/PD-L1 checkpoint blockade. If both cell types are killed by the fast mechanism, the resulting tumor on Day 25 is twenty times smaller than if both cell types are killed by the slow mechanism, as illustrated by the difference in size of the dots in the upper right and lower left corners. If high antigen tumor cells are killed only via the fast mechanism, then low antigen tumor cells must have at least 0.33 probability of being killed via the fast mechanism in order to achieve at least 75% tumor reduction on Day 25. The relative values of $p_1, p_2$ determine the prevailing type of tumor cells in the long run. When $p_1 > p_2$, checkpoint blockade therapy will increase the proportion of low antigen phenotype in the tumor despite reducing the total tumor size. This change in tumor composition will impact the tumor’s responsiveness to future treatments. For example, since low antigen tumor cells in our baseline assumption elicit slower immune response, a more low antigen-dominant tumor will likely be less responsive to subsequent immunotherapy.

Figure 3D shows that by Day 150 several $p_1, p_2$ combinations lead to clinical elimination. However, there are also $p_1, p_2$ combinations that lead to substantial tumor reduction on Day 25, but complete relapse by Day 150, e.g. $p_1 = 0.85,\, p_2 = 0.36$. It is also noteworthy that our Day 25 prediction that when $p_1 > p_2$, checkpoint blockade therapy will increase the proportion of low antigen tumor cells still holds on Day 150.

### Outcomes for virtual tumors before and after checkpoint blockade

**Figure 4:**
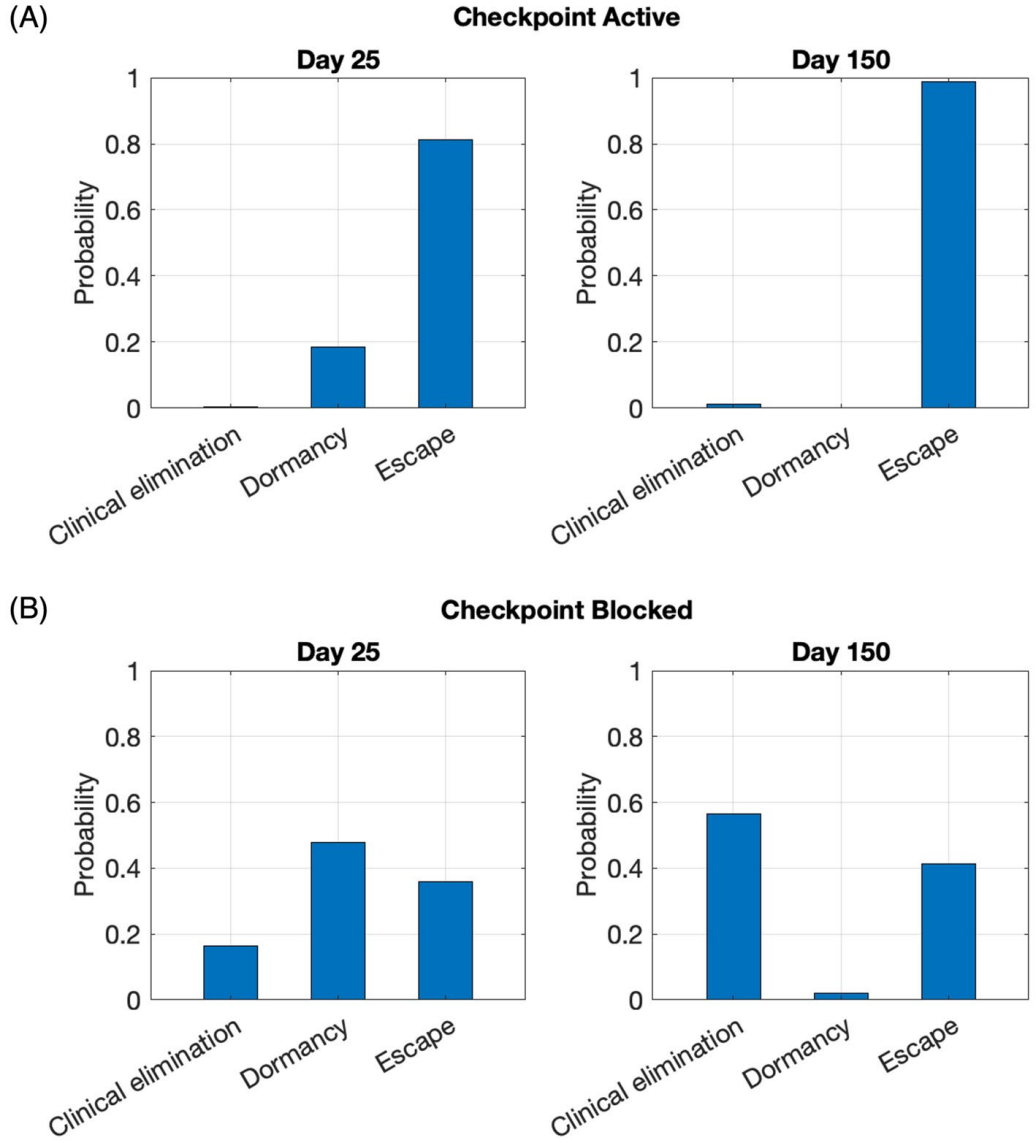
Virtual cohort simulations show that checkpoint blockade results in better clinical outcomes on both Day 25 and Day 150. (**A**) Probability of each clinical outcome when the checkpoint is active. (**B**) Probability of each clinical outcome when the checkpoint is blocked. Clinical elimination: tumor size $< 0.1 \,\text {mm}^{3}$; dormancy: $0.1 \le$ tumor size $\le 500 \,\text {mm}^{3}$, escape: $> 500 \,\text {mm}^{3}$). Size of virtual cohort: 30,000.

To further investigate the effect of PD-1/PD-L1 checkpoint blockade on tumor reduction, we generate a virtual cohort of mice upon which to test therapeutic impact. To predict the response of a diverse population comprising individuals with heterogeneous tumor and immune characteristics to checkpoint therapy, we use Latin Hypercube Sampling (LHS) to generate 30,000 combinations of all parameters, initial tumor composition and initial immune to tumor cell ratio. Each parameter combination represents a mouse with unique tumor and immune dynamics. We categorize our simulated outcomes before and after complete checkpoint blockade into clinical elimination (tumor size less than $0.1\,\text {mm}^{3}$), dormancy (tumor size between $0.1$ and $500\,\text {mm}^{3}$) and escape (greater than $500\,\text {mm}^{3}$). Figure 4A shows that when the checkpoint is active approximately 20$\%$ of tumors are small enough to be dormant on Day 25, but almost 80$\%$ are already large enough to be in the escape category. By Day 150, almost all dormant tumors have progressed to escape with only a very small fraction being clinically eliminated.

When the checkpoint is completely blocked, Fig. 4B shows that on Day 25 about 16% of tumors are clinically eliminated and only about 36% have escaped. By Day 150, most tumors that were dormant on Day 25 have been clinically eliminated and a small amount have escaped. Taken together, the results illustrated in Fig. 4 suggest that Day 25 tumor size alone can be a poor predictor of long-term clinical outcomes, especially when the tumor is small but detectable.

### Correlation between key immune parameters and therapeutic outcomes

**Figure 5:**
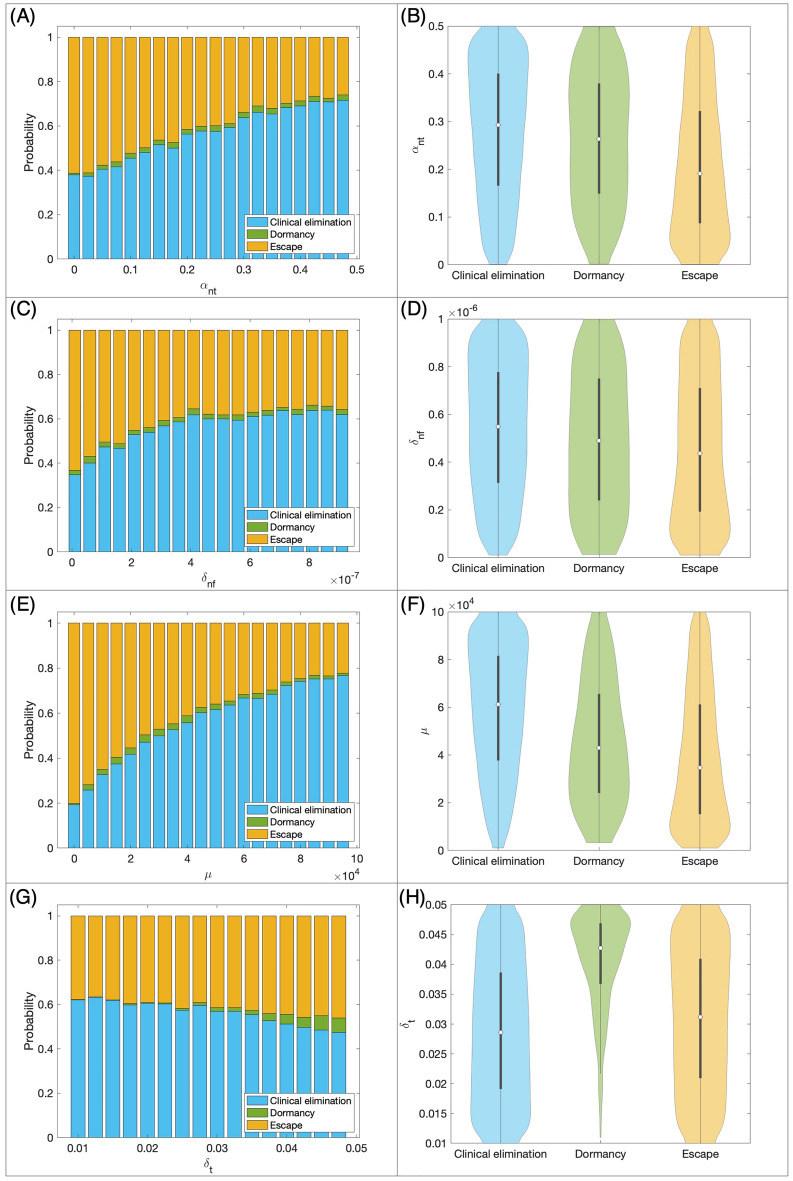
Relationship between model parameters and the outcome of checkpoint blockade therapy in the same virtual cohort as Fig. 4. (**A**,**C**,**E**,**G**) Stacked bar plots of the probability of each outcome (clinical elimination, dormancy, or escape) for a range of a chosen impactful parameter. (**B**,**D**,**F**,**H**) violin plots of the distribution of parameters of the virtual cohort associated with each outcome, with the shape showing probability density, the white circle showing median and the grey lines showing interquartile range. ($\alpha _{nt}$: maximum rate of CTL proliferation activated by high antigen tumor cells; $\delta _{nf}$: CTL-induced death rate of high antigen tumor cells via the fast-killing mechanism; $\mu$: activation and recruitment rate of T cells; $\delta _t$: death rate of T cells).

We showed in the previous section that mice with different tumor-immune landscapes have distinct responses to checkpoint therapy. In Fig. 5 we investigate how clinical outcomes depend on the values of important parameters, which relate to specific tumor and immune characteristics in our cohort of 30,000 mice. Figure 5A,C,E,G are stacked bar plots showing the probability of each outcome (clinical elimination, dormancy, or escape) for a range of a chosen parameter that impacts clinical outcome. Figure 5B,D,F,H are violin plots showing the distribution, median and interquartile range of parameters in our virtual cohort associated with each outcome.

Figure 5A implies that as the maximum rate of CTL proliferation activated by high antigen tumor cells ($\alpha _{nt}$) increases from smallest values to the largest, the probability of clinical elimination also increases from 0.38 to 0.72, while the probability of escape decreases from 0.61 to 0.26. In our cohort, the $\alpha _{nt}$ median and interquartile range for escape cases are lower than that of the clinical elimination and dormancy cases as shown in Fig. 5B. CTL-induced death rate of high antigen tumor cells via the fast-killing mechanism ($\delta _{nf}$) and activation and recruitment rate of T cells ($\mu$) show similar trends (Fig. 5C,D,E,F). However, the trend is more pronounced for $\mu$. As $\mu$ increases from the smallest to the largest values, the probability of elimination quadruples while the probability of escape  drops down to one fourth (Fig. 5E). Moreover, the $\mu$ median and interquartile range of elimination cases is also significantly higher than that of escape cases. These all reiterate the importance of $\mu$ in tumor elimination as we explained in Fig. 2. Regarding the death rate of T cells ($\delta _t$), we observe that the probability of clinical elimination decreases slightly and probability of escape increases slightly as $\delta _t$ increases (Fig. 5G). We also notice that dormancy is extremely unlikely with small $\delta _t$ and its probability improves slightly with bigger $\delta _t$ values.

## Discussion

Here we presented the first mechanistic mathematical model designed to investigate the impact on immunotherapy outcomes of differential cell-kill strategies immune cells use to target tumor cells with different antigen loads. To predict which patients are more likely to respond to ICIs, and to develop improved therapeutic strategies, understanding the differential cell-kill mechanisms that T cells use against tumor cells is essential. Since the discovery of the PD-1/PD-L1 immune checkpoint, the immunosuppressive effect of the checkpoint has been included in several mathematical models of tumor-immune dynamics^14,16–18^. While our model has similar features to existing models, our work is distinctive and innovative in two ways: (i) we consider two types of tumor cells (high antigen and low antigen phenotype), and (ii) we explicitly incorporate the two killing mechanisms.

We assessed the impact of ICI monotherapy or combination therapy by analyzing the relationship of important model parameters to tumor volume and composition. Based on bifurcation analysis of potential long-term tumor responses, we categorized therapeutic outcomes into “elimination”, “dormancy” and “escape”, which can correspond to the three phases of the immunoediting framework^21^. Other works such as^22^ have categorized outcomes of tumor-immune dynamics similarly. “Elimination” referred to a tumor volume of $0\,\text {mm}^{3}$, while “dormancy” referred to a tumor with volume less than $500\,\text {mm}^{3}$ and “escape” referred to a tumor expanding larger than $500\,\text {mm}^{3}$. To better understand how parameters impact therapy outcomes in a more clinically relevant time frame, we supplemented bifurcation analysis with direct visualization of Day 25 and Day 150 tumor volume and composition with varying parameters in Fig. 3. We chose the endpoints, Day 25 and Day 150, to reflect immediate and long-term responses to therapy. In this case, we re-coined “elimination” as “clinical elimination” to refer to a clinically undetectable tumor with volume less than $0.1\,\text {mm}^{3}$, representing the limit of clinical and imaging assessment.

A novel feature of our model was that the probability of tumor cell death via fast killing differs based on tumor antigenicity, with $p_1$ being the probability associated with high antigen tumor cells and $p_2$ associated with low antigen tumor cells. Our analysis showed that, after checkpoint blockade therapy, $p_1$ and $p_2$ critically determine whether tumor elimination is possible and how tumor composition changes if the tumor persists. In Fig. 3, we showed that if the probability of tumor cell death via fast killing is sufficiently high for both high and low antigen tumor cells, ICI monotherapy can clinically eliminate the tumor. Under our baseline assumption, high antigen tumor cells are more likely to be killed via the fast mechanism than low antigen tumor cells, i.e. $p_1 > p_2$. In this case, when the tumor persists under ICI monotherapy, the resulting tumor is more low antigen-dominant than the initial tumor in the short term. The tumor then progresses to become wholly composed of low antigen tumor cells over time. If our baseline assumption holds, our result suggested that the presence of low antigen tumor cells weakens the response to ICI monotherapy. A related phenomenon was observed clinically in a recent study by Zapata et al.^23^, which demonstrated that immune-edited tumors with low tumor antigenicity are less likely to respond to ICIs.

Furthermore, we studied how to enhance the therapeutic results of ICI with combination therapy. To do this, we focused on parameters that can be altered by current clinical interventions. CTL activation and recruitment rate ($\mu$) proved to be the most important immune parameters for achieving tumor reduction or elimination, followed by maximum rate of antigen-mediated CTL proliferation ($\alpha _{nt}, \alpha _{mt}$) . This model finding is consistent with clinical observations that have shown that activation of T cells via immune checkpoint blockade is not effective for non-T cell-inflamed tumors, which do not have T cell recruitment beyond a minimal threshold^24^. Furthermore, in preclinical models, the systemic administration of activating, proliferating tumor antigen-specific T cells is insufficient to control tumors that lack the appropriate chemokine signals for recruitment of T cells^25^. We showed that increasing $\mu$ can lead to a much smaller tumor or even elimination, with other tumor-immune characteristics kept constant. One way to increase CTL activation and recruitment is through adoptive T cell therapy. Emerging clinical evidence supports the use of adoptive cell therapy using tumor-infiltrating lymphocytes after anti-PD-1 or PD-L1 therapy^26,27^, as well as the use of CAR T-cell therapy before anti-PD-1 to treat solid tumors^28^. Moreover, according to our simulations, increasing $\alpha _{nt}$ or $\alpha _{mt}$ can also lead to significant volume reduction. IL-2, a cytokine that promotes the growth and activation of T cells, is often used in combination with other forms of immunotherapy, including adoptive T cell transfer^19^. Therefore, cytokine therapy can be potentially combined with ICIs to increase antigen-mediated T cell proliferation ($\alpha _{nt}$, $\alpha _{mt}$) and produce better therapeutic outcomes. Using a humanized mouse model, Jespersen et al. determined continuous presence of IL-2 to be essential for eradicating tumors undergoing adoptive T cell transfer and/or anti-PD-1^29^, although they did not manage to show that anti-PD-1 and adoptive T cell transfer combination therapy is superior than adoptive T cell transfer alone. In practice, IL-2 therapy combined with ICI has not proven more effective than ICI monotherapy to date^30^, although a recent phase 1b study suggested that combination therapy with high-dose IL-2 therapy and anti-PD-1 might be feasible and tolerable^31^. Nonetheless, new variants of IL-2 for synergy with ICI continued to be developed in clinical setting^30^. Overall, combination therapy may have two significant benefits: (i) achieve tumor elimination or drastic tumor volume reduction that is otherwise unattainable with ICI alone; (ii) reduce the amount of drug used in each treatment, which patients might tolerate better.

Beyond immunotherapy alone, our analysis showed that ICIs can also be combined with first-line treatments like chemotherapy to produce therapeutic benefits. The “elimination or escape” and “dormancy or elimination” bistability regions in Fig. 2 suggested that, depending on the initial tumor size, tumor composition, or ratio of tumor to immune cells, two patients with similar tumor-immune landscapes may have tumors of vastly different sizes after receiving checkpoint blockade therapy. Furthermore, mouse models showed that chemotherapies that induce immunogenic cell death can turn a non-T cell-inflamed tumor into a T cell-inflamed tumor more infiltrated with tumor-specific T cells^32^. Some chemotherapies can upregulate the expression of PD-L1 by cancer cells^32^. Since T cell-inflamed tumors elicit different immune responses from non-T cell-inflamed tumors, which would translate to different immune or tumor parameters in our model, ICI can produce qualitatively different therapeutic outcomes depending on the baseline endogenous immune response to the tumor. Therefore, chemotherapy may be used before ICI to enhance the efficacy of ICI and facilitate tumor elimination. This strategy has been successful clinically in bladder cancer where maintenance therapy with avelumab, an anti-PD-L1 therapy, has become standard of care with an overall survival benefit^33^.

To single out the impact of each aforementioned model parameter on an individual patient, we had to keep the other parameters invariant. Through virtual cohort analysis, we shifted our focus to a diverse population and study the relationship between specific characteristics of the tumor-immune landscape and the outcomes of checkpoint blockade therapy or combination therapy. High CTL recruitment rate ($\mu$), antigen-mediated T cell proliferation rates ($\alpha _{nt}, \alpha _{mt}$), and CTL-induced death rate of tumor cells via fast killing ($\delta _{nf}, \delta _{mf}$) all corresponded to a higher probability of tumor elimination after checkpoint blockade therapy. This was particularly pronounced for $\mu$: patients with a high CTL recruitment rate ($\mu$) were four times more likely to get their tumors eliminated than those with a low $\mu$. If these patient-specific parameters can be measured, they can be used to predict the probability of tumor elimination after receiving ICI alone. If other forms of cancer treatments (e.g. adoptive T cell transfer, cytokine therapy) can modulate these parameters, they should be considered for combination therapy with ICI for better odds of tumor elimination. The correlation between the rate of fast killing for high and low antigen tumor cells ($\delta _{nf}, \delta _{mf}$) and the outcomes of checkpoint blockade therapy highlighted the importance of considering differential immune cell-kill mechanisms when evaluating the efficacy of ICI. Furthermore, virtual cohort simulations suggested that it might be too soon to conclude the outcome of ICI on Day 25, because tumors that shrunk significantly by Day 25 might relapse by Day 150, as implied by results in Figs. 3 and 4.

This study thoroughly investigated the outcomes of complete immune checkpoint blockade as the best-case scenario for using ICIs by assuming that $F(P,L) = 1$. In reality, the value of *F*(*P*, *L*) will likely be less than 1 and vary with time, depending on how anti-PD1 is administered. Hence, we will implement a realistic dosing schedule of anti-PD-1 therapy in the virtual cohort in our future work to improve the clinical relevance of the model. Terms and parameters of ODE models also have to be carefully chosen to ensure that key parameters can be calibrated with real-world data. Therefore, one cannot represent all relevant reaction pathways and have to omit many details of biological processes in an ODE model. Despite the computational and analytical advantages of continuous ODEs in modeling tumor-immune dynamics, the lack of spatial components in ODEs leads to the inability to obtain structural information about the tumor and the tumor microenvironment. Agent-based models, which describe each cell as an independent agent in a three-dimensional space and prescribe how cells move or interact, can better reflect the complexity seen in vivo and complement ODE models. These approaches, along with parameterization of ODE models with in vivo data in the future, will improve representation of diverse patient populations in the models.

Data-driven and biologically informed mathematical models of cancer control strategies are a powerful complement to experimental studies. Mechanistic ODE models like the ones presented in this work allow rapid simulations to identify critical patterns or discover underlying mechanisms in the tumor microenvironment that drive cancer progression and therapeutic resistance. In particular, we explored how differential cell-kill mechanisms that T cells use against tumor cells with variable antigenicity impact tumor growth and their implications on ICI monotherapy or combination therapy. Our methodology can be used to systematically explore a wide range of questions related to tumor-immune dynamics and immunotherapy. The modeling framework and extensions proposed can provide valuable insights for the rational design of pre-clinical experiments and clinical trials.

### Supplementary Information

Supplementary Information 1.

Supplementary Information 2.

## Data Availability

The original contributions presented in the study are included in the article/supplementary material, further inquiries can be directed to the corresponding author.
