# Supplementary material for: Mathematical model predicts tumor control patterns induced by fast and slow cytotoxic T lymphocyte killing mechanisms
Source: Sci Rep. 2023 Dec 18;13:22541. doi: 10.1038/s41598-023-49467-6 (PMC10728095; doi:10.1038/s41598-023-49467-6)
Supplement: Supplementary file 1 — Supplementary Information 1. [file 41598_2023_49467_MOESM1_ESM.pdf]

Model formulation

Variable	Description	Units	Initial Condition (baseline and range)
N	Tumor cells with high antigenicity	#	5×10^5 $(0 - 10^6)$
M	Tumor cells with low antigenicity	#	5×10^5 $(0 - 10^6)$
T	Cytotoxic T Lymphocytes	#	1000 $(1 - 10^5)$

Table 1: Variables for the ODE model with active immune checkpoint. We use the conversion factor 10^6 tumor cells equal $1mm^3$ [5]. Total number of tumor cells (N+M) = 10^6 in all simulations.

We consider a heterogeneous tumor made up of tumor cells with both high and low antigenicity. Equation (1) models the temporal dynamics of the tumor cells that express high antigen levels.

$$\frac{dN}{dt} = \underbrace{\alpha_n N \left(1 - \frac{N+M}{K}\right)}_{\substack{\text{Logistic growth} \\ \text{of N cells}}} - \underbrace{\delta_{ns}(1-p_1)\frac{N}{\kappa_1 T + (N+M) + \kappa_0} T}_{\substack{\text{FasL-mediated (slow) killing} \\ \text{of N cells}}} - \underbrace{\delta_{nf} p_1 N T}_{\substack{\text{Perforin-mediated (fast)} \\ \text{killing of N cells}}} \tag{1}$$

The first term in Equation (1) describes tumor cells with high antigenicity proliferating logistically with growth rate α_n and carrying capacity K. This logistic form reflects the implicit competition for space and resources that occurs between the two cell types. The second term in Equation (1) describes the slow killing of high antigen tumor cells with probability $1 - p_1$ and maximum rate δ_{ns}. Here, slow killing is modeled by the "Beddington" function like the saturating function used in [2]. We assume that each activated CTL kills multiple tumor cells, independent of their antigenicity, primarily by inducing apoptosis in a contact dependent manner. The third term in equation (1) describes the faster, perforin-mediated killing of high antigen tumor cells with probability p_1 and maximum rate δ_{nf}. We model rapid cell killing as being directly proportional to the numbers of CTLs and tumor cells as described in [3]. There is some evidence that, with strong antigenic stimulation, there can be a substantial proportion of high-rate killer CTLs [9]. Although this could imply that high antigen tumor cells are preferentially killed via the fast mechanism, we keep the model general and assume that high antigen tumor cells can be killed via both the fast perforin-mediated pathway and the slow FasL-mediated pathway in order to test the differential impact of cell kill decisions.

Equation (2) models the temporal dynamics of the tumor cells that express low antigen levels.

$$\frac{dM}{dt} = \underbrace{\alpha_m M \left(1 - \frac{N+M}{K}\right)}_{\substack{\text{Logistic growth} \\ \text{of M cells}}} - \underbrace{\delta_{ms}(1-p_2)\frac{M}{\kappa_1 T + (N+M) + \kappa_0} T}_{\substack{\text{FasL-mediated (slow) killing} \\ \text{of M cells}}} - \underbrace{\delta_{mf} p_2 M T}_{\substack{\text{Perforin-mediated (fast)} \\ \text{killing of M cells}}} \tag{2}$$

Although quantitative insights into the dynamic cell kill patterns of CTLs are essential for the rational design of immune-based therapies, the CTL killing landscape with a growing tumor remains insufficiently characterized [1]. There is evidence that sustained killing at the population level, relies on a highly variable, multiple-killing performance at the individual CTL level [9]. Therefore, we decided to model the growth and killing of low antigen tumor cells just like the high antigen tumor cells, with necessary changes to the names of the parameters. By allowing maximum flexibility in which type of tumor cells are predominantly killed by each mechanism, we are able to investigate the impact of probabilities for fast (p_1,p_2) and slow kill ($1 - p_1$,$1 - p_2$) on the final tumor volume and

composition. However, we anticipate, based on [9], that $p_1 > p_2$, i.e. high antigen tumor cells are more likely to be killed via the fast mechanism than low antigen tumor cells.

In our model the CTLs we consider are activated CD8+ cytotoxic T cells, which are terminally differentiated effector T cells with cytotoxic activity. Equation (3) models the temporal dynamics of these activated CD8+ T cells.

$$\frac{dT}{dt} = \left(\underbrace{\mu}_{\substack{\text{Activation/} \\ \text{recruitment of T cells}}} + \underbrace{\alpha_{nt}\frac{N}{\kappa_2 + N}T + \alpha_{mt}\frac{M}{\kappa_2 + M}T}_{\substack{\text{Antigen-mediated} \\ \text{proliferation of T cells}}} \right) \cdot \underbrace{F(P,L)}_{\substack{\text{immune suppression} \\ \text{by PD-1/PD-L1}}} - \underbrace{\delta_n NT}_{\substack{\text{N cell mediated} \\ \text{T cell death}}} - \underbrace{\delta_m MT}_{\substack{\text{M cell mediated} \\ \text{T cell death}}} - \underbrace{\delta_t T}_{\substack{\text{Natural} \\ \text{T cell death}}} \tag{3}$$

The first term in (3) represents a constant recruitment/activation of T cells at rate μ. The second and third terms describes proliferation that occurs as the result of antigenic stimulation by the tumor cells. We assume both tumor phenotypes can elicit proliferation of T cells, although we anticipate $\alpha_{nt} > \alpha_{mt}$. $F(P,L)$ models the immune suppression by the PD-1/PD-L1 complex. As in [4, 5, 6, 7], the function for suppression of T cell activation and proliferation by the PD-1/PD-L1 complex is given by:
where P and L represent the concentrations of PD-1 and PD-L1 respectively. This formulation ensures that as PD-1 and PD-L1 increase, so does the number of PD-1/PD-L1 complexes. An increase in these immune checkpoint complexes corresponds to a smaller value of $F(P,L)$, thereby modeling the inhibition of T cell activity. Finally the last three terms in equation (3) describe the ways in which

$$F(P,L) = \frac{1}{1 + \frac{PL}{k_{TQ}}} \tag{4}$$

CTLs die. Specifically, interaction with high antigen expressing cells can result in death at rate δ_N, interaction with low antigen expressing cells can result in death at rate δ_M, and CTLs can die naturally at rate δ_T.

The checkpoint active model described above serves as the baseline case, which can be modulated by adjusting the immunosuppressive effects. We will consider the special case of 100% effective checkpoint blockade therapy, which corresponds to $F(P,L) = 1$ because it can provide insights about the best-case scenario outcomes for therapies targeting PD-1 or PD-L1. Baseline parameters in Table 1 are chosen from literature where possible. Due to the novel components in our model, such as different cell kill mechanisms for high and low antigen tumor phenotypes, we chose parameters not available in the literature so that the total tumor volume on Day 25 after complete checkpoint blockade is reduced by 75%, compared to the checkpoint active case.

References

[1] Richard J Beck, Dario I Bijker, and Joost B Beltman. Heterogeneous, delayed-onset killing by multiple-hitting t cells: Stochastic simulations to assess methods for analysis of imaging data. *PLOS Computational Biology*, 16(7):1–25, 2020.

[2] Harsh V Jain, IC Sorribes, SK Handelman, J Barnaby, and Trachette L Jackson. Standing variations modeling captures inter-individual heterogeneity in a deterministic model of prostate cancer response to combination therapy. *Cancersl*, 13(8):1872, 2021.

[3] Vladimir A Kuznetsov, Iliya A Makalkin, Mark A Taylor, and Alan S Perelson. Nonlinear dynamics of immunogenic tumors: parameter estimation and global bifurcation analysis. *Bulletin of mathematical biology*, 56(2):295–321, 1994.

[4] Xiulan Lai and Avner Friedman. Combination therapy of cancer with cancer vaccine and immune checkpoint inhibitors: A mathematical model. *PLoS One*, 12(5):e0178479, 2017.

[5] Ugo Del Monte. Does the cell number still really fit one gram of tumor tissue? *Cell cycle*, 8(3):505–506, 2009.

[6] Elpiniki Nikolopoulou, Lauren Johnson, Duane Harris, John Nagy, Edward Stites, and Yang Kuang. Tumour-immune dynamics with an immune checkpoint inhibitor. *Letters in Biomathematics*, 5(2):S137–S159, 2018.

[7] Kamaldeen Okuneye, Daniel Bergman, Jeffrey C Bloodworth, Alexander T Pearson, Randy F Sweis, and Trachette L Jackson. A validated mathematical model of fgfr3-mediated tumor growth reveals pathways to harness the benefits of combination targeted therapy and immunotherapy in bladder cancer. *Computational and Systems Oncology*, 1(2):e1019, 2021.

[8] Kathleen M Storey, Sean E Lawler, and Trachette L Jackson. Modeling oncolytic viral therapy, immune checkpoint inhibition, and the complex dynamics of innate and adaptive immunity in glioblastoma treatment. *Frontiers in physiology*, 11:151, 2020.

[9] Zilton Vasconcelos, Sabina Mu¨ller, Delphine Guipouy, Wong Yu, Claire Christophe, S´ebastien Gadat, Salvatore Valitutti, and Lo”ic Dupr´e. Individual human cytotoxic t lymphocytes exhibit intraclonal heterogeneity during sustained killing. *Cell Reports*, 11(9):1474–1485, 2015.
