# Supplementary material for: Mathematical model predicts tumor control patterns induced by fast and slow cytotoxic T lymphocyte killing mechanisms
Source: Sci Rep. 2023 Dec 18;13:22541. doi: 10.1038/s41598-023-49467-6 (PMC10728095; doi:10.1038/s41598-023-49467-6)
Supplement: Supplementary file 2 — Supplementary Information 2. [file 41598_2023_49467_MOESM2_ESM.pdf]

Sensitivity of tumor growth to parameters characterizing the tumor-immune landscape

Many details of tumor-immune interactions in the presence or absence of the PD-1/PD-L1 immune checkpoint remain unknown or hard to quantify, leading to uncertainty in model parameters and initial conditions. We use global sensitivity analysis to understand the impact of this uncertainty and to determine which parameters have the greatest impact on tumor growth when the PD-1/PDL1 immune checkpoint is active and when it is completely blocked. Following [1], we assess global sensitivity by using Latin hypercube sampling (LHS) and the partial rank correlation coefficient (PRCC) between the parameters and the tumor volume on Day 25 and Day 150. We used 30000 sample sets of parameters and determined it was sufficient because the PRCC values had stabilized even if sample size increased further. Sensitive parameters are defined to be those in the upper quartile of all PRCC values in terms of magnitude and with a p-value of less than 0.05. Figure S1(A) shows the PRCC between the parameters and the tumor volume when the checkpoint is active. The tumor volume is most sensitive to α_n, α_m, K. On Day 25, most tumors are still growing and have not reached the carrying capacity. Therefore, tumor proliferation rates (α_n, α_m) have higher PRCC value on Day 25 than Day 150. With checkpoint active, most tumors have reached the carrying capacity on Day 150, which explains the nearly perfect positive correlation between tumor volume and the carrying capacity (K).

When the PD-1/PD-L1 checkpoint is blocked, there is a different set of sensitive parameters as the PRCC results in Figure S1(B) shows. The total tumor volume is still sensitive to α_n, α_m (tumor proliferation rates) but less sensitive to K (carrying capacity). When the checkpoint is blocked, the total tumor volumes are in general smaller and well below the carrying capacity, resulting in the smaller PRCC value for K. The tumor volume is now most sensitive to μ (activation and recruitment rate of T cells), followed by κ_2 (half-saturation constant in antigen mediated T cell proliferation), α_{nt}, α_{mt} (maximum rate of antigen-mediated CTL proliferation), α_n, α_m, δ_{nf}, δ_{mf} (CTL-induced death rate via fast killing), K and δ_t (death rate of T cells). The total tumor volume is negatively correlated with α_{nt}, α_{mt}, δ_{nf}, δ_{mf}, μ and positively correlated with κ_2, α_n, α_m, K, δ_t. The results show that any therapy that enhances antigen-independent T cell recruitment, increases the rates of either type of killing mechanism, or increases antigen-mediated T cell proliferation may be combined with immune checkpoint blockade therapy to effectively reduce tumor size.

We conducted similar analysis (Figure S2) to determine which parameters have the greatest impact on tumor composition, which we measure by the ratio of low antigen tumor cells to total tumor cells. The endpoint of PRCC analysis is now low antigen tumor cells to total tumor cells ratios on Day 25 and Day 150. In this case, with checkpoint blocked or active, ratio of low antigen to total tumor cell is sensitive to the same set of variables: α_n, α_m, δ_{nf}, δ_{ns}, p_1 (probability of high antigen cell death via fast killing), p_2 (probability of low antigen cell death via fast killing) and R_{LA} (initial ratio of low antigen to total tumor cells). In particular, p_1 and p_2 are new sensitive parameters that are important mediators of the ratio of low antigen tumor cells to total tumor cells.

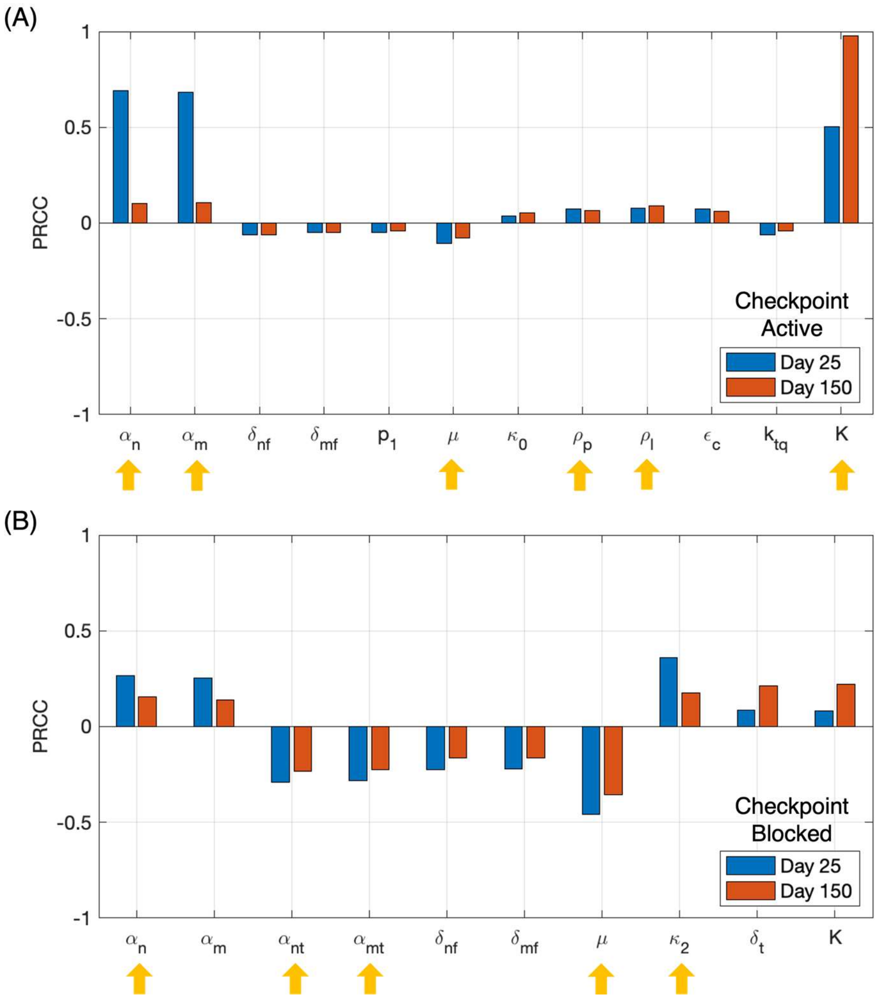

Figure S1: Immune checkpoint activity affects the most sensitive parameters with respect to tumor volume. (A) PRCCs (partial rank correlation coefficient) of parameters in the model with immune checkpoint active. (B) PRCCs of parameters in the model with immune checkpoint blocked. Blue: PRCC with respect to total tumor volume on Day 25. Red: PRCC with respect to total tumor volume on Day 150. Yellow arrows: sensitive parameters with magnitude of PRCC ranked in the top quartile. Parameters with magnitude of PRCC ranked in the top 50% are shown.

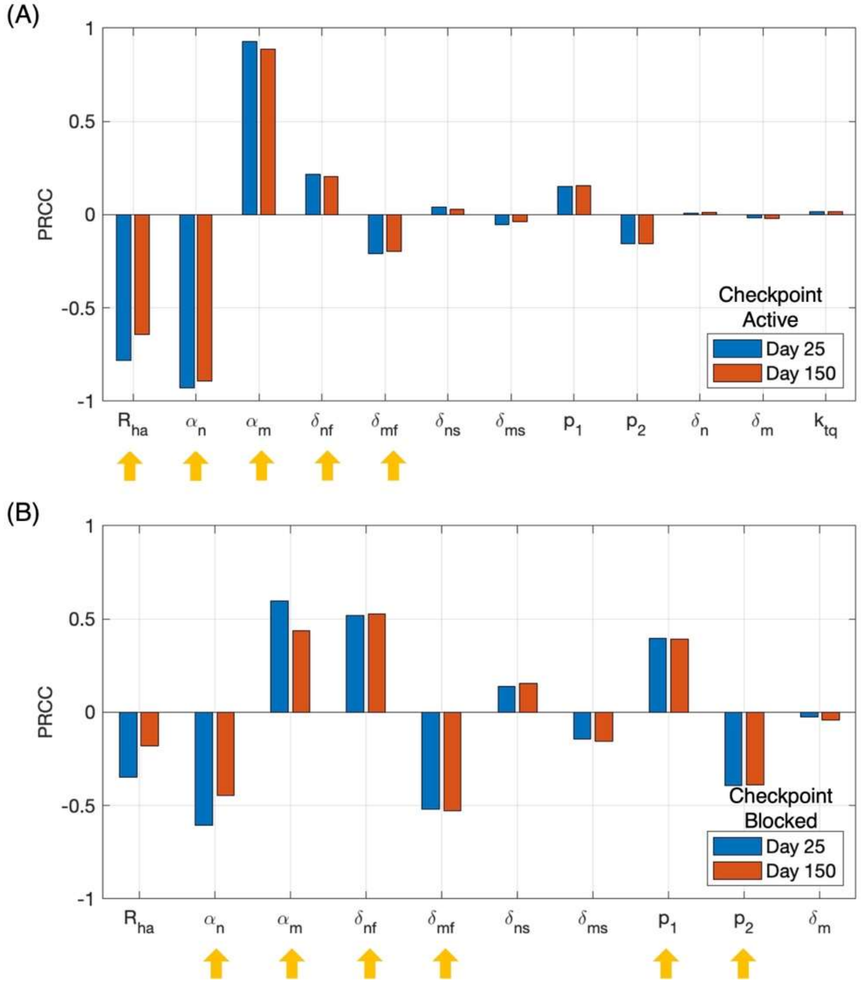

Figure S2: Immune checkpoint activity affects the most sensitive parameters with respect to ratio of low antigen tumor cells to total tumor cells. (A), (B) see Figure S1.

References

[1] Kamaldeen Okuneye, Daniel Bergman, Jeffrey C Bloodworth, Alexander T Pearson, Randy F Sweis, and Trachette L Jackson. A validated mathematical model of fgfr3-mediated tumor growth reveals pathways to harness the benefits of combination targeted therapy and immunotherapy in bladder cancer. *Computational and Systems Oncology*, 1(2):e1019, 2021.
